# Phase angle as a predictor of mortality in elderly patients with multimorbidity: a matched case-control study

**DOI:** 10.7717/peerj.18592

**Published:** 2024-11-26

**Authors:** Yao Wang, Tingting Liu, Tianru Zheng, Yun Zhang, Li Li, Maolong Gao

**Affiliations:** 1 Elderly Demonstration Ward, Beijing Geriatric Hospital, Beijing, China; 2 Beijing lnstitute of Integrative Medicine Geriatrics, Beijing Geriatric Hospital, Beijing, China

**Keywords:** Phase angle, Bioelectrical impedance analysis, Elderly, Multimorbidity, Mortality

## Abstract

**Background:**

We aimed to investigate the value of phase angle (PhA) as a prognostic marker for mortality in elderly patients with multimorbidity using a matched case-control study.

**Methods:**

This study was conducted with patients 70 years of age or older with multimorbidity at Beijing Geriatric Hospital. The body composition parameters including PhA were determined using bioelectrical impedance analysis. Common hematological indices were determined using blood tests. The outcome was mortality 1 year after admission. A multivariate logistic regression analysis was employed to identify independent risk factors for death. A receiver-operating characteristic (ROC) curve analysis was used to evaluate the performance of risk factors in predicting death.

**Results:**

A total of 30 deceased patients were included in the death group. The living patients were matched 1:1 with the deceased patients in age, gender, and Cumulative Illness Rating Scale-Geriatric score to generate a survival group of 30. The death group exhibited higher levels of blood urea nitrogen and extracellular water to total body water ratio and lower levels of PhA and prealbumin than the survival group. The multivariate logistic regression analysis identified PhA as the only independent risk factor for mortality (OR = 3.296, 95% CI [1.201–9.044], *P* < 0.05). For the ROC curve analysis, PhA had an area of 0.854 (95% CI [0.755–0.955], *P* = 000). The Youden index was 0.700, and the optimal cutoff value associated with the Youden index was 2.45°.

**Conclusion:**

PhA serves as a good prognostic marker for mortality in elderly patients with multimorbidity.

## Introduction

Multimorbidity refers to the presence of two or more long-term health conditions. Older adults are highly susceptible to multimorbidity of aging-related chronic diseases such as cancer, atherosclerosis, diabetes, and cognitive decline. Studies have found that 65% of 65–84 years old and 82% of 85 years or older suffer from multimorbidity, making medical care for elderly patients both challenging and complex (Barnett et al., 2012; Fortin et al., 2012). Another characteristic of elderly patients is their altered nutritional status. Aging is often associated with changes in food intake, nutrient absorption, and physical activity, and these changes may lead to malnutrition (Newberry & Dakin, 2021). Additionally, an elderly patient’s nutritional status can deteriorate due to disease-related wasting and drug-related side-effects (Schuetz et al., 2021). Notably, 60–80% of elderly patients with cancer or other chronic wasting diseases have a poor nutritional status, which is associated with increased clinical complications, prolonged hospital stay, and poor prognosis (Bellanti et al., 2022). Hence, accurate assessment of patient nutritional status is critical for improving medical care of frail elderly patients, especially those with multimorbidity.

Malnutrition in the elderlies is often manifested as altered body composition characterized by progressive loss of lean muscle mass (sarcopenia) and accumulation of body fat. Bioelectrical impedance analysis (BIA) is a noninvasive, inexpensive, and reproducible bedside method for assessing body composition by sending a weak current through the body. As different body compartments (*e.g*., muscle, fat, bone, *etc*.) have different electrical conductivity due to their varying water content, BIA can estimate body composition parameters such as muscle mass and fat mass. As such, BIA is widely used in various clinical settings for nutritional assessment and management (Brantlov et al., 2017a, 2017b; Guo et al., 2023; Kyle et al., 2004; Sbrignadello, Gobl & Tura, 2022; Ward, 2019). Phase angle (PhA) is a BIA-derived parameter that reflects cell integrity and water distribution within and outside the cell. It is calculated as the ratio Xc/R in degrees (o), where resistance (R) measures how easily electrical currents pass through tissues, and capacitive reactance (Xc) represents the cells’ ability to store and release energy (Ward & Brantlov, 2023). Low PhA is associated with frailty in the general population (Tanaka et al., 2019). In many pathophysiological conditions, PhA has demonstrated usefulness as a prognostic marker for mortality (Bellido et al., 2023). In addition, PhA decreases with age, and elderly people with low PhA have a high risk of death (Kwon et al., 2023; Wilhelm-Leen et al., 2014).

In this work, we used a matched case-control study to investigate the relationship between PhA and mortality in elderly patients with multimorbidity. This study was conducted at Beijing Geriatric Hospital in Beijing, China.

## Materials and Methods

### Patients

This was a matched case-control study. Patients with multimorbidity who were ≥70 years old and admitted to Beijing Geriatric Hospital (Beijing, China) between January 1, 2020 and May 31, 2022 were subjected to BIA within 72 h of admission. The patients underwent routine blood tests on the same day of BIA. Co-morbidities were evaluated using the Cumulative Illness Rating Scale-Geriatric (CIRS-G) within 24 h of admission. Multimorbidity was diagnosed based on the CIRS-G score (Inciong et al., 2020). The follow-up time for death or survival was 1 year. Patients who had any of the following conditions and thus were not suitable for BIA measurements were excluded: pacemaker or defibrillator implantation, body or limb abnormalities, significant edema or massive ascites, or skin damage at BIA electrode contact sites. Patients who died from sudden unexpected events such as car accidents or trauma during the follow-up period were also excluded. This study was approved by the Ethics Committee of Beijing Geriatric Hospital (Beijing, China) (approval number 2018BJLNYY-2018-008) and was performed in compliance with the Declaration of Helsinki. All participants provided written informed consent.

### BIA measurements

The principle of BIA is to treat the human body as a conductive cylinder, run a weak electric current through the body, and measure impedance, which comprises both resistance (R) and reactance (Xc). R reflects the opposition to the flow of the current, which is mainly determined by the amount of intracellular and extracellular water. Xc indicates the capacitive losses caused by cell membranes. Different body components have different levels of impedance in response to varying electrical frequencies. Body composition parameters are estimated based on BIA measurements using empirical regression equations. In particular, PhA, which reflects cell membrane integrity and function, is calculated from R and Xc using the equation: PhA = arctangent (Xc/R) × (180°/π) (Kushner, 1992).

The patients were prohibited from diuretics use within 48 h and food, water, shower, and vigorous exercise within 2 h before BIA measurements. They were asked to empty the bladder and lie flat in bed for at least 30 min prior to BIA. BIA was performed in the supine position on a Inbody S10 device (Inbody, Seoul, Korea). Body composition parameters including fat mass (FM), fat-free mass (FFM), skeletal muscle mass (SMM), body mass index (BMI), visceral fat area (VFA), basal metabolic rate (BMR), body cell mass (BCM), intracellular water (ICW), extracellular water (ECW), total body water (TBW), ratio of ECW to TBW (ECW/TBW), and PhA were calculated based on the height, weight, and impedance data acquired. All parameters were determined using the in-build equations of the InBody device.

### Blood tests

Hematological indices including lymphocyte count, hemoglobin, lipids (triglycerides and cholesterol), markers of nutritional status (serum albumin and prealbumin), and markers of renal function (blood urea nitrogen (BUN) and serum creatinine) were determined by blood test on an Abbott ARCHITECT ci16200 Integrated System on the same day of BIA measurement.

### Statistically analysis

In a matched case-control study, the patients were divided into two groups, death or survival, based on their status at the end of follow-up. The survival patients were matched 1:1 with the death patients in age, gender, and CIRS-G score. The death patients and matching survival patients were included in statistical analysis. The data were analyzed using SPSS.25 (IBM, Armonk, NY, USA). Continuous variables are presented as mean ± SD (standard deviation) and were compared using the independent samples *t*-test and analysis of variance (ANOVA). Discrete variables are presented as mean ± sem (standard error of the mean) and were compared using the *t* test. The normality of the data was checked using distribution histogram in SPSS. A *P* value less than 0.05 was considered statistically significant. The logistic regression analysis was employed to identify independent risk factors for death. The receiver-operating characteristic (ROC) curve analysis was used to evaluate the performance of the risk factors in predicting death.

## Results

### Demographics and CIRS-G scores

A total of 121 patients who were diagnosed with multimorbidity and met the other inclusion criteria underwent BIA evaluation. The patients were 82.5 ± 8.1 years old on average. A total of 30 patients had died at the end of follow-up (1 year after admission) and were included in the death group. The patients who were still alive at the end of follow-up were matched 1:1 with the death patients in age, gender, and CIRS-G score to generate a survival group of 30 patients. Table 1 summarizes the comparison of baseline data between the death and survival groups. There were no significant differences in the age, male/female ratio, or multimorbidity burden between the two groups.

**Table 1:** Comparison of baseline data between the death and survival groups.

	Death (*n* = 30)	Survival (*n* = 30)	T-value	*P*-value
Age (years)	85.6 ± 9.5	85.4 ± 6.0	0.097	0.923
Gender	Male 15, female 15	Male 14, female 16		
CIRS-G score	14.8 ± 3.0	12.4 ± 2.8	1.644	0.104

**Note:**

Data are presented as mean ± SD. T-value, *t*-test value; *P*-value, probability.

### Body composition parameters and hematological indices

Table 2 summarizes the comparison of the BIA-derived body composition parameters and hematological indices between the death and survival groups. Significant differences in ECW/TBW, PhA, and serum prealbumin and BUN levels were detected between the death and survival groups (*P* < 0.05). Compared with the survival group, the death group exhibited higher BUN and ECW/TBW and lower PhA and prealbumin. In particular, the death group showed an PhA of (2.34 ± 0.92)°, significantly lower than that of the survival group, which was (3.73 ± 1.47)° (*P* = 000). No significant differences in other body composition parameters or hematological indices were detected between the two groups.

**Table 2:** Comparison of body composition parameters and hematological indices between the death and survival groups.

	Death (*n* = 30)	Survival (*n* = 30)	T-value	*P*-value
TBW (L)	29.6 ± 9.0	28.4 ± 7.6	0.552	0.583
ICW (L)	17.2 ± 5.5	17.0 ± 4.8	0.172	0.864
ECW (L)	12.4 ± 3.5	11.4 ± 2.9	1.147	0.256
ECW/TBW	0.42 ± 0.03	0.40 ± 0.21	2.865	0.006
FFM (kg)	39.8 ± 12.1	38.5 ± 10.2	−0.242	0.809
FM (kg)	19.1 ± 11.1	20.2 ± 6.0	−0.486	0.629
VFA (cm^2^)	127.6 ± 83.1	117.8 ± 50.6	0.552	0.583
SMM (kg)	20.5 ± 7.2	20.2 ± 6.2	0.180	0.857
BMI (kg/m^2^)	21.7 ± 5.4	22.5 ± 3.4	−0.686	0.496
BMR (kcal/(h · kg))	1231 ± 262	1201 ± 23	0.467	0.642
BCM (kg)	24.7 ± 7.9	24.3 ± 6.8	0.183	0.855
PhA (°)	2.34 ± 0.92	3.73 ± 1.47	−4.393	0.000
Lymphocyte count (10^9^ cells/L)	1.28 ± 0.69	1.52 ± 0.68	−1.31	0.194
Hemoglobin (g/L)	106.8 ± 24.1	110.8 ± 26.2	−6.22	0.537
Albumin (g/L)	33.8 ± 5.4	37.2 ± 3.8	−1.132	0.262
Prealbumin (mg/L)	158.6 ± 80.0	187.9 ± 53.9	−2.875	0.006
Triglyceride (mmol/L)	1.61 ± 1.13	1.49 ± 0.99	0.433	0.667
Cholesterol (mmol/L)	6.73 ± 6.80	3.79 ± 0.75	0.958	0.342
BUN (mg/dL)	12.59 ± 7.04	7.24 ± 4.14	3.588	0.001
Creatinine (µmol/L)	126.2 ± 150.6	79.6 ± 55.6	1.589	0.118

**Note:**

Data are presented as mean ± SD. T-value, *t*-test value; *P*-value, probability.

### Multivariate logistic regression analysis for identifying independent risk factors for death

To identify independent risk factors for mortality, a multivariate logistic regression analysis was carried out using the four death-associated factors (ECW/TBW, PhA, serum prealbumin, and BUN). The results are presented in Table 3. PhA was identified as the only independent risk factor for mortality (OR = 3.296, 95% CI [1.201–9.044], *P* < 0.05). Table 4 presents the relationship between PhA and mortality. ECW/TBW, serum prealbumin, and BUN showed no independent association with death (*P* > 0.05).

**Table 3:** Multivariate logistic regression analysis for identifying independent risk factors for death.

	B-value	SE B	Wald X^2^	*P*-value	OR	95% CI OR
ECW/TBW	−28.09	34.17	0.676	0.411	0.000	[0.000–7.649]
PhA	1.193	0.515	5.364	0.021	3.296	[1.201–9.044]
Prealbumin	0.003	0.005	0.314	0.575	1.003	[0.993–1.013]
BUN	−0.131	0.071	3.405	0.065	0.877	[0.763–1.008]

**Note:**

B-value, regression coefficient B; SE B, standard error of B; *P*-value, hypothesis test of the regression coefficient B-value; Wald X^2^, Wald chi-square distribution value; OR, odds ratio; 95% CI, 95% confidence interval.

**Table 4:** The relationship between PhA and mortality.

	PhA ≤ 1°	1° < PhA ≤ 2°	2° < PhA ≤ 3°	3° < PhA ≤ 4°	4° < PhA ≤ 5°	PhA > 5°
Dead (*n*)	2	11	11	4	2	0
Total (*n*)	2	11	32	34	26	16
Mortality	100%	100%	34.4%	11.8%	7.7%	0

### ROC curve analysis of PhA in predicting death

**Figure 1:**
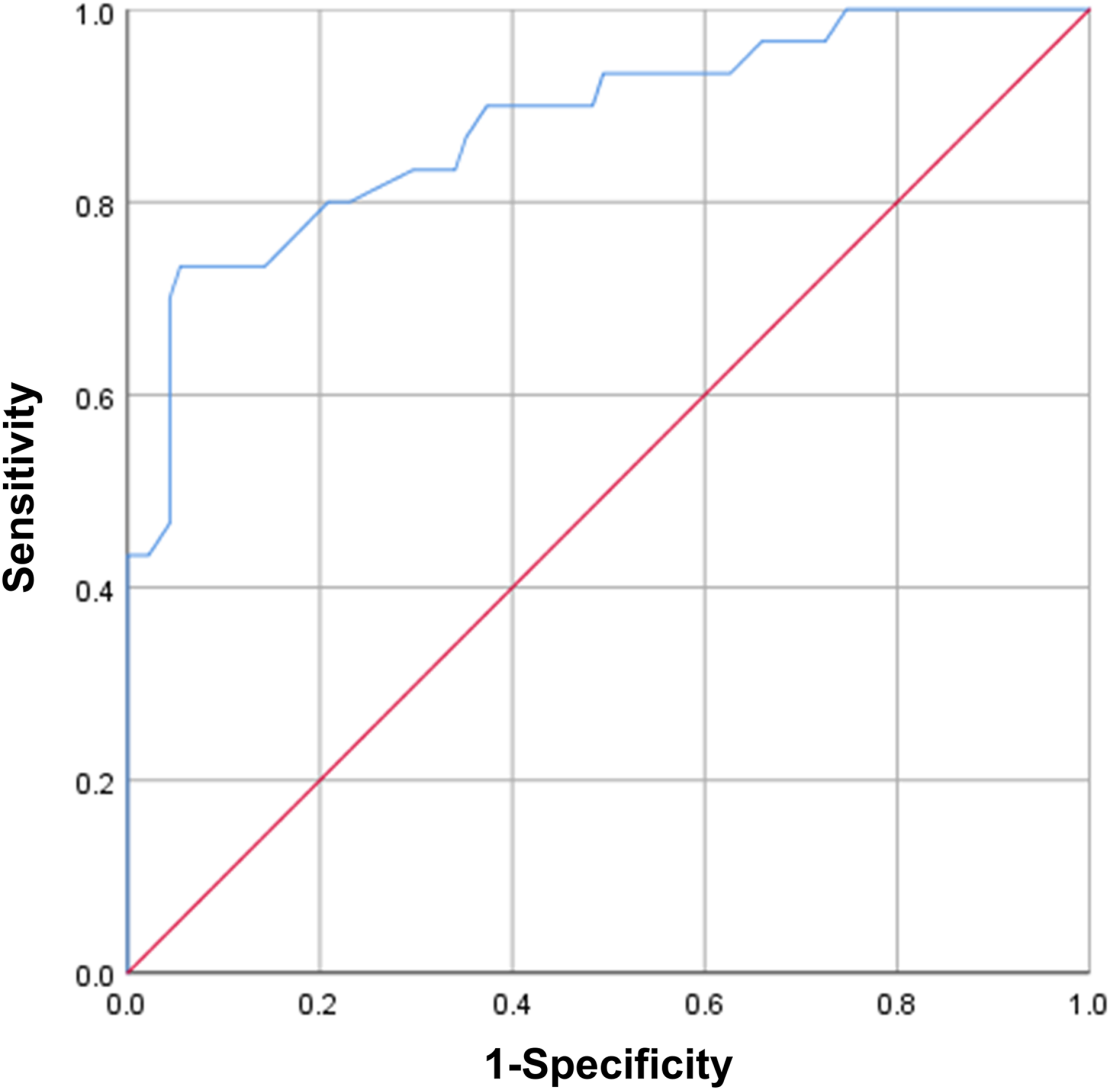
ROC curve analysis of PhA in predicting mortality.

Next, we conducted ROC curve analysis of PhA to evaluate its performance in predicting mortality in elderly patients with multimorbidity. Figure 1 presents the ROC curve analysis of PhA. PhA showed an area of 0.881 (95% CI [0.805–0.957], *P* = 0000). The Youden index was 0.678, and the optimal cutoff value associated with the Youden index was 2.55°.

## Discussion

This matched case-control study identified PhA as an independent risk factor for mortality in elderly patients with multimorbidity. Thus, PhA may serve as a good prognostic marker in this specific patient population. Malnutrition is a very common health concern in older adults, because people in this age group often have not only declined food intake and nutrition adsorption but also aging-related chronic diseases, which can aggravate the nutritional problem. Malnutrition causes frailty and weakens immunity in elderly patients, and as such, it may contribute to the development of geriatric syndromes and/or exasperate existing chronic disorders, leading to prolonged hospital stay and increased morbidity and mortality (Norman, Hass & Pirlich, 2021). Malnutrition can be difficult to diagnose due to the lack of a clear definition of the disorder and standard methods for evaluating nutritional status. Detection of malnutrition in elderly patients with multimorbidity is particularly challenging, because these patients often have complex multiorgan dysfunctions that result in various abnormal blood test results. Serum albumin and hemoglobin have traditionally been used as biomarkers of patient nutritional status (Farag et al., 2023; Smith, 2017). However, abnormal levels of albumin and hemoglobin can result from inflammation (Don & Kaysen, 2004; Wang, Du & Zhu, 2020), which occurs frequently in elderly patients. Thus, common hematological indices have limited value as biomarkers for assessing the nutritional status of elderly patients, especially those with multimorbidity.

Lukaski et al. (1985) first pioneered the use of BIA as a method for the analysis of body composition in 1985. Since then, BIA has been widely used as a bedside tool for the assessment of patient nutritional status in many diseases, wherein malnutrition may be either a cause, such as sarcopenia, or a consequence, such as cancer (Earthman, 2015). Compared with other BIA-derived body composition parameters such as ICW and ECW, PhA is less influenced by body fluid distribution. Higher PhA value reflect greater cellular integrity and function, and hence better nutritional status, while low PhA values indicate impaired cellular health and poor nutritional status. Adult patients between 17 and 65 years of age in a US hospital had a significantly lower PhA than age- and height-matched healthy controls (male 6.6 *vs*. 7.6, *P* < 0.001; female 5.8 *vs*. 6.5, *P* < 0.001) (Kyle et al., 2012), indicating that PhA is a general indicator of health. PhA has demonstrated usefulness as a diagnostic or prognostic marker under many pathophysiological conditions. In critically ill patients such as those with severe COVID-19, low PhA is associated with prolonged intensive care unit (ICU) stay and increased risk of mortality and hence the need for more supportive care (Cornejo-Pareja et al., 2022a, 2022b; Lima et al., 2022). Malnutrition is a very common complication of cancer, with a reported prevalence of up to 80% among cancer patients (Sonmez et al., 2022). It can be caused by the malignancy itself or result from conventional cancer treatments. Studies of PhA in cancer have revealed significant associations of PhA with nutritional status, physical function, quality of life, complications, and patient survival (Arab et al., 2021). However, PhA cutoff values vary significantly according to the study design, type of cancer, and patient demographics (*e.g*., age and gender). As such, these factors should be taken into account when PhA is used to detect malnutrition in cancer patients. In patients with digestive or liver diseases, PhA serves as an indicator of disease stage and severity, as well as response to interventions (Casirati et al., 2023). Lean soft tissue mass is an indicator of muscle strength and exercise capacity in patients with chronic lung diseases such as chronic obstructive pulmonary disease (COPD). PhA allows clinical assessment of lean soft tissue mass in these patients (De Benedetto, Marinari & De Blasio, 2023). In patients with metabolic diseases such as obesity, PhA can be used as a tool for monitoring weight loss and body composition improvements following dietary, physical, or pharmacological therapies (Cancello et al., 2023). PhA research is critical for the development of new approaches for the treatment of obesity and related diseases. It is particularly useful for establishing long-term weight maintenance programs after weight loss (Cancello et al., 2023). Patients with chronic kidney disease (CKD) are overloaded with fluid and at high risk for malnutrition due to protein energy wasting, which can lead to worsened renal function and increased mortality risk (Maggiore et al., 1996). PhA can be used, along with other BIA parameters, as a clinical tool to evaluate the nutritional and fluid status of CKD patients for treatment decision making. In particular, it can be used for volume management during hemodialysis to minimize adverse intradialytic responses (Kotanko, 2019). Heart failure can disturb the normal function of the kidney and impair sodium excretion from the body, resulting in water retention. PhA is associated with BIA parameters of water load and distribution such as TBW/FFM and ECW/TBW and therefore, it may be used in the clinical evaluation, management, and prognostic stratification of patients with acute or chronic heart failure (Liang et al., 2021). Protein-energy malnutrition is often observed in older adults, which leads to sarcopenia, the aging-related loss of muscle mass and strength (Sieber, 2019). PhA is positively associated with muscle mass and strength in older adults and therefore, it may be considered a good BIA marker for assessing the risk of sarcopenia in hospitalized elderly patients (Santana et al., 2018).

This study was the first investigation on the potential value of PhA in predicting death among hospitalized elderly patients with multimorbidity. We found that, in this patient population, ECW/TBW and BUN were positively associated with death, while PhA and prealbumin showed an inverse correlation. ECW/TBW, BUN, PhA, and prealbumin are all biomarkers used for health evaluation and clinical outcome prediction in certain patient populations. ECW/TBW is an indicator of edema and is associated with severity of nutritional status (Ge et al., 2022). BUN is the major product of protein metabolism in the human body and is mainly excreted by the kidneys. High BUN levels indicate impaired renal function and poor protein metabolism (Park et al., 2021). Prealbumin, a hepatic protein, is a nutritional and prognostic marker in patients who are critically ill or have a chronic disease (Beck & Rosenthal, 2002). In this study, a multivariate logistic regression analysis using the four death-associated factors (ECW/TBW, PhA, prealbumin, and BUN) identified PhA as the only independent risk factor for mortality (OR = 3.296, 95% CI [1.201–9.044], *P* < 0.05). The death group showed an PhA of (2.34 ± 0.92)°, significantly lower than that of the survival group, which was (3.73 ± 1.47)° (*P* = 000). The ROC curve analysis of PhA revealed an area of 0.881 (95% CI [0.805–0.957], *P* = 000), a Youden index of 0.678, and an optimal cutoff value of 2.55°, suggesting that PhA has good sensitivity and specificity in predicting death in elderly patients with multimorbidity. However, this study was limited by a small sample size, which included only 30 death and 30 survival elderly patients with multimorbidity. Moreover, this was a single-center study, which may limit the generalizability of the findings. Further research in larger and more diverse populations, along with considering other potential confounding variables, would be necessary to solidify its significance in the field of clinical prognostication.

The growing burden of disease-specific multimorbidity in the elderlies is becoming a global health problem, especially in countries where the population is rapidly ageing, such as Japan and China. Geriatric patients vary in cognitive, physical, and social functions, and thus need special care based on individual needs and disease conditions. To develop an appropriate plan of care for each individual patient, a team of healthcare professionals needs to conduct a comprehensive geriatric assessment, which is a multidimensional evaluation of the medical and functional status of patients, as well as their overall well-being. This process is time consuming and can be difficult due to staffing shortages or lack of training. BIA is a noninvasive, convenient, and inexpensive bedside tool that provides useful information on patient nutritional status and overall well-being. When used in combination with hematological indices, BIA can enable early detection and thus early intervention of malnutrition in geriatric patients, thereby improving patient prognosis and quality of life.

To date, more than 50% of PhA research has been conducted in Europe, followed by the US, Brazil, Japan, and China (Bellido et al., 2023). Currently, BIA is available in some but not all hospitals in China, and it is used mostly for the assessment of nutritional status and body composition of obese patients (Fu et al., 2022). Coordinated efforts are required to implement this technology in geriatric hospitals in China for improving patient evaluation and personalized care.

## Conclusions

Phase angle serves as an independent prognostic marker for mortality in elderly patients with multimorbidity. It can be used for early detection of malnutrition in geriatric patients, enabling early intervention and improving patient prognosis and quality of life.

## Supplemental Information

10.7717/peerj.18592/supp-1 Supplemental Information 1 Raw data of phase angle in participants.

10.7717/peerj.18592/supp-2 Supplemental Information 2 STROBE checklist.

## Additional Information and Declarations

### Data Availability

The following information was supplied regarding data availability: The raw data of phase angle in participants are available in the Supplemental File 1.
