# Supplementary material for: Phase angle as a predictor of mortality in elderly patients with multimorbidity: a matched case-control study
Source: PeerJ. 2024 Nov 26;12:e18592. doi: 10.7717/peerj.18592 (PMC11606320; doi:10.7717/peerj.18592)
Supplement: Supplemental Information 2 [file peerj-12-18592-s002.docx]

STROBE Statement—checklist of items that should be included in reports of observational studies

|  | Item No. | Recommendation | Page  No. | Relevant text from manuscript |
| --- | --- | --- | --- | --- |
| **Title and abstract** | 1 | (*a*) Indicate the study’s design with a commonly used term in the title or the abstract | 1 | A matched case-control study to evaluate phase angle as a marker of mortality risk in elderly patients with multimorbidity |
|  |  | (*b*) Provide in the abstract an informative and balanced summary of what was done and what was found | 2 | The body composition parameters including PhA were determined using bioelectrical impedance analysis. Common hematological indices were determined using blood tests. The outcome was mortality one year after admission. A multivariate logistic regression analysis was employed to identify independent risk factors for death. A receiver-operating characteristic (ROC) curve analysis was used to evaluate the performance of risk factors in predicting death. A total of 30 deceased patients were included in the death group. The living patients were matched 1:1 with the deceased patients in age, gender, and Cumulative Illness Rating Scale-Geriatric score to generate a survival group of 30. The death group exhibited higher levels of blood urea nitrogen and extracellular water to total body water ratio and lower levels of PhA and prealbumin than the survival group. The multivariate logistic regression analysis identified PhA as the only independent risk factor for mortality (OR = 3.296, 95% CI 1.201–9.044, P < 0.05). For the ROC curve analysis, PhA had an area of 0.854 (95% CI 0.755–0.955, P = 000). The Youden index was 0.700, and the optimal cutoff value associated with the Youden index was 2.45°. In conclusion, PhA serves as a good prognostic marker for mortality in elderly patients with multimorbidity. |
| Introduction | | | |  |
| Background/rationale | 2 | Explain the scientific background and rationale for the investigation being reported | 3-4 | Multimorbidity refers to the presence of two or more long-term health conditions. Older adults are highly susceptible to multimorbidity of aging-related chronic diseases such as cancer, atherosclerosis, diabetes, and cognitive decline. Studies have found that 65% of 65–84 years old and 82% of 85 years or older suffer from multimorbidity, making medical care for elderly patients both challenging and complex *(Barnett et al., 2012; Fortin et al., 2012)*. Another characteristic of elderly patients is their altered nutritional status. Aging is often associated with changes in food intake, nutrient absorption, and physical activity, and these changes may lead to malnutrition *(Newberry and Dakin, 2021)*. Additionally, an elderly patient's nutritional status can deteriorate due to disease-related wasting and drug-related side-effects *(Schuetz et al., 2021)*. Notably, 60–80% of elderly patients with cancer or other chronic wasting diseases have a poor nutritional status, which is associated with increased clinical complications, prolonged hospital stay, and poor prognosis *(Bellanti et al., 2022)*. Hence, accurate assessment of patient nutritional status is critical for improving medical care of frail elderly patients, especially those with multimorbidity.  Malnutrition in the elderlies is often manifested as altered body composition characterized by progressive loss of lean muscle mass (sarcopenia) and accumulation of body fat. Bioelectrical impedance analysis (BIA) is a noninvasive, inexpensive, and reproducible bedside method for assessing body composition by running a weak current through the body. As different body compartments (e.g. muscle, fat, bone, etc.) have different electrical conductivity due to their varying water content, BIA can estimate body composition parameters such as muscle mass and fat mass. As such, BIA is widely used in various clinical settings for nutritional assessment and management *(Guo et al., 2023; Sbrignadello et al., 2022)*. Phase angle (PhA) is a BIA-derived body composition parameter that reflects cell integrity and water distribution within and outside the cell *(Ward and Brantlov, 2023)*. Low PhA is associated with frailty in the general population *(Tanaka et al., 2019)*. In many pathophysiological conditions, PhA has demonstrated usefulness as a prognostic marker for mortality *(Bellido et al., 2023)*. In addition, PhA decreases with age, and elderly people with low PhA have a high risk of death *(Kwon et al., 2023; Wilhelm-Leen et al., 2014)*. |
| Objectives | 3 | State specific objectives, including any prespecified hypotheses | 4 | In this work, we used a matched case-control study to investigate the relationship between PhA and mortality in elderly patients with multimorbidity. This study was conducted at Beijing Geriatric Hospital in Beijing, China. |
| Methods | | | |  |
| Study design | 4 | Present key elements of study design early in the paper | 5 | In a matched case-control study, the patients were divided into two groups, death or survival, based on their status at the end of follow-up. The survival patients were matched 1:1 with the death patients in age, gender, and CIRS-G score. |
| Setting | 5 | Describe the setting, locations, and relevant dates, including periods of recruitment, exposure, follow-up, and data collection | 4 | Patients with multimorbidity who were ≥70 years old and admitted to Beijing Geriatric Hospital (Beijing, China) between January 1, 2020 and May 31, 2022 were subjected to BIA within 72 hours of admission. Co-morbidities were evaluated using the Cumulative Illness Rating Scale-Geriatric (CIRS-G) within 24 hours of admission. Multimorbidity was diagnosed based on the CIRS-G score (Inciong et al., 2020). The follow-up time was 1 year. |
| Participants | 6 | (*a*) *Cohort study*—Give the eligibility criteria, and the sources and methods of selection of participants. Describe methods of follow-up  *Case-control study*—Give the eligibility criteria, and the sources and methods of case ascertainment and control selection. Give the rationale for the choice of cases and controls  *Cross-sectional study*—Give the eligibility criteria, and the sources and methods of selection of participants | 4-5 | Patients with multimorbidity who were ≥70 years old and admitted to Beijing Geriatric Hospital (Beijing, China) between January 1, 2020 and May 31, 2022 were subjected to BIA within 72 hours of admission. The patients underwent routine blood tests on the same day of BIA. Co-morbidities were evaluated using the Cumulative Illness Rating Scale-Geriatric (CIRS-G) within 24 hours of admission. Multimorbidity was diagnosed based on the CIRS-G score (Inciong et al., 2020). The follow-up time was 1 year. Patients who had any of the following conditions were excluded: pacemaker or defibrillator implantation, body or limb abnormalities, significant edema or massive ascites, or skin damage at BIA electrode contact sites.  In a matched case-control study, the patients were divided into two groups, death or survival, based on their status at the end of follow-up. The survival patients were matched 1:1 with the death patients in age, gender, and CIRS-G score. |
|  |  | (*b*) *Cohort study*—For matched studies, give matching criteria and number of exposed and unexposed  *Case-control study*—For matched studies, give matching criteria and the number of controls per case | 5 | In a matched case-control study, the patients were divided into two groups, death or survival, based on their status at the end of follow-up. The survival patients were matched 1:1 with the death patients in age, gender, and CIRS-G score. |
| Variables | 7 | Clearly define all outcomes, exposures, predictors, potential confounders, and effect modifiers. Give diagnostic criteria, if applicable | 4 | Co-morbidities were evaluated using the Cumulative Illness Rating Scale-Geriatric (CIRS-G) within 24 hours of admission. Multimorbidity was diagnosed based on the CIRS-G score (Inciong et al., 2020). |
| Data sources/ measurement | 8* | For each variable of interest, give sources of data and details of methods of assessment (measurement). Describe comparability of assessment methods if there is more than one group | 4-5 | BIA measurements  Diuretics use within 48 hours and food, water, shower, and vigorous exercise within 2 hours before measurement were prohibited. The patients were asked to empty the bladder and lie flat in bed for at least 30 minutes prior to BIA. BIA was performed in the supine position on a Inbody S10 device (Inbody, Korea). Body composition parameters including fat mass (FM), fat-free mass (FFM), skeletal muscle mass (SMM), body mass index (BMI), visceral fat area (VFA), basal metabolic rate (BMR), body cell mass (BCM), intracellular water (ICW), extracellular water (ECW), total body water (TBW), ratio of ECW to TBW (ECW/TBW), and PhA were calculated based on the height, weight, and impedance data acquired.  Blood tests  Blood tests determined hematological indices including lymphocyte count, hemoglobin, lipids (triglycerides and cholesterol), markers of nutritional status (serum albumin and prealbumin), and markers of renal function (blood urea nitrogen (BUN) and serum creatinine). |
| Bias | 9 | Describe any efforts to address potential sources of bias | NA |  |
| Study size | 10 | Explain how the study size was arrived at | NA |  |

Continued on next page

| Quantitative variables | 11 | Explain how quantitative variables were handled in the analyses. If applicable, describe which groupings were chosen and why | 5 | In a matched case-control study, the patients were divided into two groups, death or survival, based on their status at the end of follow-up. The survival patients were matched 1:1 with the death patients in age, gender, and CIRS-G score. |
| --- | --- | --- | --- | --- |
| Statistical methods | 12 | (*a*) Describe all statistical methods, including those used to control for confounding | 5 | The data were analyzed using SPSS 25. Continuous variables are presented as mean ± SD (standard deviation) and were compared using the independent samples t-test and analysis of variance (ANOVA). Discrete variables are presented as mean ± sem (standard error of the mean) and were compared using the t test. A P value less than 0.05 was considered statistically significant. The logistic regression analysis was employed to identify independent risk factors for death. The receiver-operating characteristic (ROC) curve analysis was used to evaluate the performance of the risk factors in predicting death. |
|  |  | (*b*) Describe any methods used to examine subgroups and interactions | NA |  |
|  |  | (*c*) Explain how missing data were addressed | NA |  |
|  |  | (*d*) *Cohort study*—If applicable, explain how loss to follow-up was addressed  *Case-control study*—If applicable, explain how matching of cases and controls was addressed  *Cross-sectional study*—If applicable, describe analytical methods taking account of sampling strategy | 5 | The survival patients were matched 1:1 with the death patients in age, gender, and CIRS-G score |
|  |  | (*e*) Describe any sensitivity analyses | NA |  |
| Results | | | | |
| Participants | 13* | (a) Report numbers of individuals at each stage of study—eg numbers potentially eligible, examined for eligibility, confirmed eligible, included in the study, completing follow-up, and analysed | 6 | A total of 121 patients who were diagnosed with multimorbidity and met the other inclusion criteria underwent BIA evaluation. The patients were 81.9±9.3 years old on average. A total of 30 patients had died at the end of follow-up (1 year after admission) and were included in the death group. The patients who were still alive at the end of follow-up were matched 1:1 with the death patients in age, gender, and CIRS-G score to generate a survival group of 30 patients. As shown in Table 1, there were no significant differences in the age, male/female ratio, or multimorbidity burden between the death and survival groups. |
|  |  | (b) Give reasons for non-participation at each stage | NA |  |
|  |  | (c) Consider use of a flow diagram | NA |  |
| Descriptive data | 14* | (a) Give characteristics of study participants (eg demographic, clinical, social) and information on exposures and potential confounders | 6 | A total of 121 patients who were diagnosed with multimorbidity and met the other inclusion criteria underwent BIA evaluation. The patients were 81.9±9.3 years old on average. A total of 30 patients had died at the end of follow-up (1 year after admission) and were included in the death group. The patients who were still alive at the end of follow-up were matched 1:1 with the death patients in age, gender, and CIRS-G score to generate a survival group of 30 patients. As shown in Table 1, there were no significant differences in the age, male/female ratio, or multimorbidity burden between the death and survival groups.  The BIA-derived body composition parameters and hematological indices of patients from the death and survival groups are summarized in Table 2. Significant differences in ECW/TBW, PhA, and serum prealbumin and BUN levels were detected between the death and survival groups (P < 0.05). Compared with the survival group, the death group exhibited higher BUN and ECW/TBW and lower PhA and prealbumin. No significant differences in other body composition parameters or hematological indices were detected between the two groups. |
|  |  | (b) Indicate number of participants with missing data for each variable of interest | NA |  |
|  |  | (c) *Cohort study*—Summarise follow-up time (eg, average and total amount) | NA |  |
| Outcome data | 15* | *Cohort study*—Report numbers of outcome events or summary measures over time | NA |  |
|  |  | *Case-control study—*Report numbers in each exposure category, or summary measures of exposure | 6 | The BIA-derived body composition parameters and hematological indices of patients from the death and survival groups are summarized in Table 2. Significant differences in ECW/TBW, PhA, and serum prealbumin and BUN levels were detected between the death and survival groups (P < 0.05). Compared with the survival group, the death group exhibited higher BUN and ECW/TBW and lower PhA and prealbumin. No significant differences in other body composition parameters or hematological indices were detected between the two groups. |
|  |  | *Cross-sectional study—*Report numbers of outcome events or summary measures | NA |  |
| Main results | 16 | (*a*) Give unadjusted estimates and, if applicable, confounder-adjusted estimates and their precision (eg, 95% confidence interval). Make clear which confounders were adjusted for and why they were included | 6-7 | To identify independent risk factors for mortality, a multivariate logistic regression analysis was carried out using the four death-associated factors (ECW/TBW, PhA, serum prealbumin, and BUN). The results are presented in Table 3. PhA was identified as the only independent risk factor for mortality (OR = 3.296, 95% CI 1.201–9.044, P < 0.05). ECW/TBW, serum prealbumin, and BUN showed no independent association with death (P > 0.05). |
|  |  | (*b*) Report category boundaries when continuous variables were categorized | 6 | The BIA-derived body composition parameters and hematological indices of patients from the death and survival groups are summarized in Table 2. Significant differences in ECW/TBW, PhA, and serum prealbumin and BUN levels were detected between the death and survival groups (P < 0.05). Compared with the survival group, the death group exhibited higher BUN and ECW/TBW and lower PhA and prealbumin. No significant differences in other body composition parameters or hematological indices were detected between the two groups. |
|  |  | (*c*) If relevant, consider translating estimates of relative risk into absolute risk for a meaningful time period | 6-7 | To identify independent risk factors for mortality, a multivariate logistic regression analysis was carried out using the four death-associated factors (ECW/TBW, PhA, serum prealbumin, and BUN). The results are presented in Table 3. PhA was identified as the only independent risk factor for mortality (OR = 3.296, 95% CI 1.201–9.044, P < 0.05). ECW/TBW, serum prealbumin, and BUN showed no independent association with death (P > 0.05). Next, we conducted ROC curve analysis of PhA to evaluate its performance in predicting mortality in elderly patients with multimorbidity. The results are presented in Figure 1. PhA showed an area of 0.881 (95% CI 0.805–0.957, P = 0000). The Youden index was 0.678, and the optimal cutoff value associated with the Youden index was 2.55°. |

Continued on next page

| Other analyses | 17 | Report other analyses done—eg analyses of subgroups and interactions, and sensitivity analyses | NA |  |
| --- | --- | --- | --- | --- |
| Discussion | | | | |
| Key results | 18 | Summarise key results with reference to study objectives | 10 | This study investigated the potential value of BIA-derived body composition parameters and common hematological indices in predicting death among hospitalized elderly patients with multimorbidity. We found that, in this patient population, ECW/TBW and BUN were positively associated with death, while PhA and prealbumin showed an inverse correlation. ECW/TBW, BUN, PhA, and prealbumin are all biomarkers used for health evaluation and clinical outcome prediction in certain patient populations.  In this study, a multivariate logistic regression analysis using the four death-associated factors (ECW/TBW, PhA, prealbumin, and BUN) identified PhA as the only independent risk factor for mortality (OR = 3.296, 95% CI 1.201–9.044, *P* < 0.05). |
| Limitations | 19 | Discuss limitations of the study, taking into account sources of potential bias or imprecision. Discuss both direction and magnitude of any potential bias | 11 | However, this study was limited by a small sample size, which included only 30 death and 30 survival elderly patients with multimorbidity. Further research in larger and more diverse populations, along with considering other potential confounding variables, would be necessary to solidify its significance in the field of clinical prognostication. |
| Interpretation | 20 | Give a cautious overall interpretation of results considering objectives, limitations, multiplicity of analyses, results from similar studies, and other relevant evidence | 10-11 | This study investigated the potential value of BIA-derived body composition parameters and common hematological indices in predicting death among hospitalized elderly patients with multimorbidity. We found that, in this patient population, ECW/TBW and BUN were positively associated with death, while PhA and prealbumin showed an inverse correlation. ECW/TBW, BUN, PhA, and prealbumin are all biomarkers used for health evaluation and clinical outcome prediction in certain patient populations. ECW/TBW is an indicator of edema and is associated with severity of nutritional status (Ge et al., 2022). BUN is the major product of protein metabolism in the human body and is mainly excreted by the kidneys. High BUN levels indicate impaired renal function and poor protein metabolism (Park et al., 2021). Prealbumin, a hepatic protein, is a nutritional and prognostic marker in patients who are critically ill or have a chronic disease (Beck and Rosenthal, 2002). In this study, a multivariate logistic regression analysis using the four death-associated factors (ECW/TBW, PhA, prealbumin, and BUN) identified PhA as the only independent risk factor for mortality (OR = 3.296, 95% CI 1.201–9.044, P < 0.05). The death group showed an PhA of (2.34±0.92), significantly lower than that of the survival group, which was (3.73±1.47) (P = 000). The ROC curve analysis of PhA revealed an area of 0.881 (95% CI 0.805–0.957, P = 000), a Youden index of 0.678, and an optimal cutoff value of 2.55°, suggesting that PhA has good sensitivity and specificity in predicting death in elderly patients with multimorbidity. However, this study was limited by a small sample size, which included only 30 death and 30 survival elderly patients with multimorbidity. Further research in larger and more diverse populations, along with considering other potential confounding variables, would be necessary to solidify its significance in the field of clinical prognostication. |
| Generalisability | 21 | Discuss the generalisability (external validity) of the study results | 11 | The growing burden of disease-specific multimorbidity in the elderlies is becoming a global health problem, especially in countries where the population is rapidly ageing, such as Japan and China. Geriatric patients vary in cognitive, physical, and social functions, and thus need special care based on individual needs and disease conditions. To develop an appropriate plan of care for each individual patient, a team of healthcare professionals needs to conduct a comprehensive geriatric assessment, which is a multidimensional evaluation of the medical and functional status of patients, as well as their overall well-being. This process is time consuming and can be difficult due to staffing shortages or lack of training. BIA is a noninvasive, convenient, and inexpensive bedside tool that provides useful information on patient nutritional status and overall well-being. When used in combination with hematological indices, BIA can enable early detection and thus early intervention of malnutrition in geriatric patients, thereby improving patient prognosis and quality of life.  To date, more than 50% of PhA research has been conducted in Europe, followed by the US, Brazil, Japan, and China (Bellido et al., 2023). Currently, BIA is available in some but not all hospitals in China, and it is used mostly for the assessment of nutritional status and body composition of obese patients (Fu et al., 2022). Coordinated efforts are required to implement this technology in geriatric hospitals in China for improving patient evaluation and personalized care. |
| Other information | |  | | |
| Funding | 22 | Give the source of funding and the role of the funders for the present study and, if applicable, for the original study on which the present article is based | NA |  |

*Give information separately for cases and controls in case-control studies and, if applicable, for exposed and unexposed groups in cohort and cross-sectional studies.

**Note:** An Explanation and Elaboration article discusses each checklist item and gives methodological background and published examples of transparent reporting. The STROBE checklist is best used in conjunction with this article (freely available on the Web sites of PLoS Medicine at http://www.plosmedicine.org/, Annals of Internal Medicine at http://www.annals.org/, and Epidemiology at http://www.epidem.com/). Information on the STROBE Initiative is available at www.strobe-statement.org.
